# Associations of mRNA:microRNA for the Shared Downstream Molecules of EGFR and Alternative Tyrosine Kinase Receptors in Non-small Cell Lung Cancer

**DOI:** 10.3389/fgene.2016.00173

**Published:** 2016-10-13

**Authors:** Fengfeng Wang, Fei Meng, Lili Wang, S. C. Cesar Wong, William C. S. Cho, Lawrence W. C. Chan

**Affiliations:** ^1^Department of Health Technology and Informatics, The Hong Kong Polytechnic UniversityHong Kong, Hong Kong; ^2^Department of Clinical Oncology, Queen Elizabeth HospitalHong Kong, Hong Kong

**Keywords:** microRNA, EGFR, alternative tyrosine kinase receptors, multiple linear regression, support vector regression model, non-small cell lung cancer

## Abstract

Lung cancer is the top cancer killer worldwide with high mortality rate. Majority belong to non-small cell lung cancers (NSCLCs). The epidermal growth factor receptor (EGFR) has been broadly explored as a drug target for therapy. However, the drug responses are not durable due to the acquired resistance. MicroRNAs (miRNAs) are small non-coding and endogenous molecules that can inhibit mRNA translation initiation and degrade mRNAs. We wonder if some downstream molecules shared by EGFR and the other tyrosine kinase receptors (TKRs) further transduce the signals alternatively, and some miRNAs play the key roles in affecting the expression of these downstream molecules. In this study, we investigated the mRNA:miRNA associations for the direct EGFR downstream molecules in the EGFR signaling pathway shared with the other TKRs, including c-MET (hepatocyte growth factor receptor), Ron (a protein tyrosine kinase related to c-MET), PDGFR (platelet-derived growth factor receptor), and IGF-1R (insulin-like growth factor receptor-1). The multiple linear regression and support vector regression (SVR) models were used to discover the statistically significant and the best weighted miRNAs regulating the mRNAs of these downstream molecules. These two models revealed the similar mRNA:miRNA associations. It was found that the miRNAs significantly affecting the mRNA expressions in the multiple regression model were also those with the largest weights in the SVR model. To conclude, we effectively identified a list of meaningful mRNA:miRNA associations: phospholipase C, gamma 1 (PLCG1) with miR-34a, phosphoinositide-3-kinase, regulatory subunit 2 (PIK3R2) with miR-30a-5p, growth factor receptor-bound protein 2 (GRB2) with miR-27a, and Janus kinase 1 (JAK1) with miR-302b and miR-520e. These associations could make great contributions to explore new mechanism in NSCLCs. These candidate miRNAs may be regarded as the potential drug targets for treating NSCLCs with acquired drug resistance.

## Introduction

Lung cancer is the top cancer killer worldwide with high mortality rate (Wang et al., 2015). Over 80% of cases belong to non-small cell lung cancer (NSCLC) with the continuously rising incidence rate every year (Al-Saleh et al., 2012). The 5-year overall survival rate of NSCLC is very low, due to lack of the effective diagnosis and treatment methods (Indovina et al., 2011). Hence, novel and effective targets are needed urgently for cancer diagnosis and therapy. The epidermal growth factor receptor (EGFR) has been broadly explored as a drug target for treatment, since it is overexpressed in various tumors, such as breast and lung carcinomas (Kim et al., 2001; Camp et al., 2005). The receptor tyrosine kinase inhibitors (TKIs), including Gefitinib and Erlotinib, are small molecules that inhibit EGFR phosphorylation, receptor activation and the subsequent signal transduction (Camp et al., 2005). However, the drug responses are not durable due to the acquired resistance. The secondary mutation in EGFR exon 20 (T790M) was found in approximately 50% of resistant tumors (Zhang et al., 2012; Kumarakulasinghe et al., 2015). Besides the mutation, activation of alternative tyrosine kinase receptors (TKRs) sharing similar downstream pathways with EGFR has been proved to be one of the multiple resistance mechanisms, including c-MET (hepatocyte growth factor receptor), Ron (a protein tyrosine kinase related to c-MET), PDGFR (platelet-derived growth factor receptor), and IGF-1R (insulin-like growth factor receptor-1) (Camp et al., 2005).

MicroRNAs (miRNAs) are small non-coding and endogenous molecules with 19–22 nucleotides, which inhibit mRNA translation initiation and degrade mRNAs (Zandberga et al., 2013). MiRNAs play a vital role in cancer development and progression by regulating the signaling circuits and the dysregulation in a cell (Zandberga et al., 2013). MiRNAs are regarded as the important biomarkers for cancer, since their expression profiles are mostly tissue- specific and able to signify human cancers (Lu et al., 2005). It has been found that nearly all the cancers have different miRNA expression profiles compared with the adjacent normal tissues, especially in lung and breast cancers (Zhang and Farwell, 2008). MiRNAs were also reported to be related to tumor invasion and metastasis (Ma et al., 2007). Let-7 is a notable miRNA in lung cancer, which is involved in the pathogenesis and significantly decreased in lung cancer tissues (Zhang and Farwell, 2008). MiR-128b directly targets EGFR, and its expression is positively correlated with clinical response and survival for Gefitinib treatment in lung cancer (Weiss et al., 2008; Lin et al., 2010). Multiple genes in the EGFR signaling pathway are targeted by miR-7, such as EGFR and protein kinase B (Akt) (Webster et al., 2009). MiRNAs are vital in the EGFR signaling pathway, and remarkable for potential drug discovery.

Due to the critical regulatory role of miRNAs in the cellular processes and disease pathology, many prediction algorithms have been made publicly online to predict the putative mRNA:miRNA pairs based on the sequence analysis, such as TargetScan and PicTar (Krek et al., 2005; Agarwal et al., 2015). However, the identification of the regulatory miRNA and mRNA pairs is still a major challenge, because a single miRNA may target several mRNAs, and multiple miRNAs may co-regulate the same mRNA (Jayaswal et al., 2011). Moreover, the prediction databases do not take into account miRNA/mRNA expression levels. The regulatory effects of miRNAs may vary in different situations. In other words, different cancer cells may have different regulatory miRNAs. Hence, Microarray profiling of miRNAs and mRNAs has been widely used to analyze the whole genome to further explore their regulatory effects. Both mRNA and miRNA expression levels can be quantified using microarray technology. We introduced multiple linear regression and support vector regression models to identify the regulatory effects of multiple miRNAs on the same mRNAs by analyzing the microarray data.

The aim of this study was to identify the direct downstream proteins of EGFR in the EGFR signaling pathway shared with the other TKRs (c-MET, Ron, PDGFR, and IGF-1R), and to further explore the candidate miRNAs affecting the mRNA expressions of these proteins. We hypothesize that there are some important downstream proteins shared by EGFR and the other TKRs, possibly leading to the resistance of EGFR TKIs, and some key miRNAs targeting these proteins are dysregulated in NSCLCs. In order to support our hypotheses, we proposed a novel mRNA:miRNA association identification approach that consists of two steps. The first step involved the identification of putative miRNA and mRNA pairs from the prediction databases. The second step is to identify the statistically significant and the best weighted miRNAs for the mRNAs of the shared downstream molecules using multiple linear regression and support vector regression models based on the microarray expression profiles. Our approach could reduce the intricate interactions between miRNAs and mRNAs to a few meaningful mRNA:miRNA associations, which facilitates the subsequent biological analysis and more efficient experimental validation. These candidate miRNAs may be served as potential drug targets for the treatment of NSCLCs with drug resistance.

## Methods

### Microarray expression data

In this study, the microarray datasets GSE51852 and GSE51853 for mRNAs and miRNAs were collected from Gene Expression Omnibus (GEO) repository database (Arima et al., 2014). Both collected mRNA and miRNA data have been normalized. The subjects were recruited in Japan. The researchers analyzed the global expression profiles of miRNAs and the target mRNAs based on a systems biology-based approach using a Bayesian network and non-parametric regression to identify the functional gene regulatory circuitry involved in the lung adenocarcinoma (Arima et al., 2014). We chose 46 subtype NSCLC specimens for the analysis of miRNA and mRNA expression profiles obtained from the same patients (Table S1). In the microarray data, each mRNA is usually interrogated by more than one probes. We took the average of the multiple probes for the same mRNA (Breslin et al., 2005; Kapp et al., 2006).

### MiRNA profiling for the shared downstream molecules

The directly interacting downstream molecules of EGFR in the EGFR signaling pathway were selected using MetaCore® from GeneGo Inc. MetaCore® incorporates the online validated interactions from researchers to the pathways. The signaling pathways of the other TKRs (c-MET, Ron, PDGFR, and IGF-1R) were also considered to select the shared downstream molecules. The miRNAs regulating the mRNAs of the shared downstream molecules were selected using the representative resources: MicroCosm-Targets (Griffiths-Jones et al., 2008), TargetScan (Agarwal et al., 2015), miRanda-mirSVR (Betel et al., 2010), miRDB (Wong and Wang, 2015), and PicTar (Krek et al., 2005). We searched for the miRNAs targeting each mRNA using these databases, and selected the miRNAs with the support of at least two out of the five databases. We further obtained the expression levels of these mRNAs and miRNAs from the microarray data. This set of mRNAs and miRNAs, as well as their expression profiles were considered for multiple regression analysis.

### Identification of statistically significant miRNAs from multiple linear regression model

After obtaining the expression of the selected mRNAs and miRNAs, we performed the multiple linear regression analysis to explore the relationship of each mRNA with the corresponding miRNAs (Wang et al., 2014a,b). Since multiple miRNAs may simultaneously target one mRNA during the post-transcriptional process, and the effect of each miRNA may be different, multiple linear regression analysis was applied to explore their relationship in formula (1):

(1) $$y=a_{0}+a_{1}\cdot x_{1}+a_{2}\cdot x_{2}+\ldots +a_{n}\cdot x_{n}$$

where *y* was the expression level of an mRNA; *x*_1_, *x*_2_, …and *x*_*n*_ were the expression profiles of the corresponding miRNAs. The parameters, *a*_0_, *a*_1_, *a*_2_, …and *a*_*n*_, represented the regression constant and coefficients, respectively.

### Support vector regression (SVR) model

Furthermore, support vector regression (SVR) machine (Smola and Vapnik, 1997; Gunn, 1998) was used to model the relationship of the selected mRNAs and multiple miRNAs. This problem was considered as approximating the set of data,

$$\left(y_{1},x_{1}\right)\text{​},\ldots \left(y_{m},x_{m}\right)\text{​},x\in R^{n},y\in R,$$

with a linear function

(2) f​(x,w)=w​·​x+b

where *f* was an output variable, which denoted the expression level of mRNA, **x** was an input feature vector, which represented the expression profiles of multiple miRNAs, and **w** and *b* were the linear regression coefficients. To obtain the model, the error was measured based on the ϵ−insensitivity loss function defined by

(3) $$L\text{​}\left(y,f\left(\text{x},\text{w}\right)\right)=\left|y-f\text{​}\left(\text{x},\text{w}\right)\right|=\{\begin{array}{c} 0,\;if\left|y-f\text{​}\left(\text{x},\text{w}\right)\right|\leq \epsilon \\ \left|y-f\text{​}\left(\text{x},\text{w}\right)\right|-\epsilon ,\;otherwise \end{array}$$

The optimal regression function can be obtained by minimizing under constraints

(4) $$R_{w,\;L}=\frac{1}{2}||\text{w}|{|}^{2}+C\left(\sum_{i\;=\;1}^{m}\varepsilon_{i}+\sum_{i\;=\;1}^{m}\varepsilon_{i}^{*}\right),$$

under constraints

$$\begin{array}{l} y_{i}-\left(wx_{i}+b\right)\leq \epsilon +\varepsilon_{i}\\ \left(wx_{i}+b\right)-y_{i}\leq \epsilon +\varepsilon_{i}^{*}\\ \;\varepsilon_{i}\geq 0\\ \;\varepsilon_{i}^{*}\geq 0 \end{array}$$

where

|y−f​(x,w)|−ϵ=ε for the data above ϵ tube

$$\left|y-f\text{​}\left(\text{x},\text{w}\right)\right|-\epsilon =\varepsilon^{*}\text{ for the data below }\epsilon \text{ tube}$$

In formula (4), the first term measured the confidence interval, the second term was used to measure the empirical risk, and C was applied to control the magnitude of approximation errors. In the simulation, C was equal to the difference between the maximum and the minimum of mRNA expression profiles.

## Results

### Selection of direct downstream molecules of EGFR shared with the other TKRs and the corresponding miRNAs

**Figure 1:**
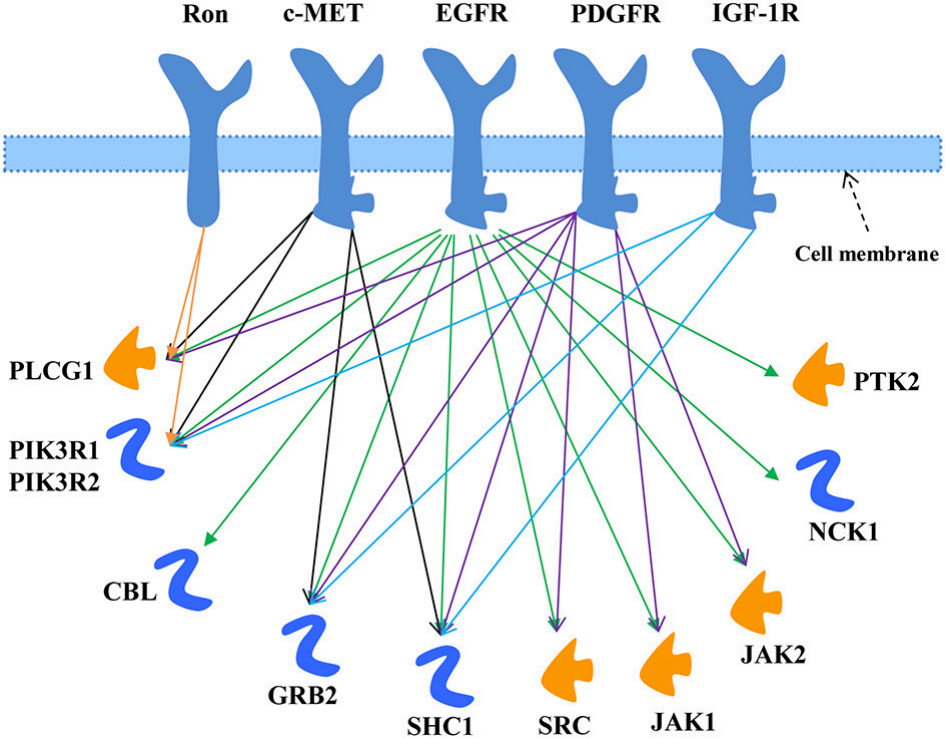
**EGFR signaling pathway modified from MetaCore®**. The downstream molecules shared with the other TKRs (c-MET, Ron, PDGFR, and IGF-1R) were marked by arrowed lines with different colors.

The EGFR signaling pathway was downloaded from MetaCore®, as shown in Figure 1. There were 11 key directly interacting downstream molecules of EGFR: phospholipase C, gamma 1 (PLCG1), phosphoinositide-3-kinase, regulatory subunit 1 & 2 (PIK3R1/2), Cbl proto-oncogene, E3 ubiquitin protein ligase (CBL), growth factor receptor-bound protein 2 (GRB2), Src homology 2 domain containing transforming protein 1 (SHC1), v-src sarcoma (Schmidt-Ruppin A-2) viral oncogene homolog (avian) (SRC), Janus kinase 1 & 2 (JAK1/2), NCK adaptor protein 1 (NCK1), and protein tyrosine kinase 2 (PTK2). In total, there were 8 out of 11 downstream molecules shared by EGFR and the other TKRs according to their signaling pathways from MetaCore® (Table 1, Figure 1). MiRNAs regulating these mRNAs were obtained from five miRNA prediction databases. The candidate miRNAs were selected, as shown in Table S2. The expression profiles of the mRNAs and the corresponding miRNAs were further extracted from the microarray datasets for regression analysis (Table S1).

**Table 1:** **Direct downstream molecules of EGFR shared with the other TKRs**.

**Shared downstream molecules**	**Shared with the other TKRs**
PLCG1	c-MET, Ron, PDGFR
PIK3R1	c-MET, Ron, PDGFR, IGF-1R
PIK3R2	c-MET, Ron, PDGFR, IGF-1R
GRB2	c-MET, PDGFR, IGF-1R
SHC1	c-MET, PDGFR, IGF-1R
SRC	PDGFR
JAK1	PDGFR
JAK2	PDGFR

### Multiple linear regression model to identify the statistically significant miRNAs regulating the shared downstream molecules

The associations of each mRNA transcript of the shared downstream molecules with the corresponding miRNAs were investigated by multiple linear regression model. This analysis was performed using IBM SPSS and MATLAB. The results demonstrated the significant miRNAs (*p* < 0.05): miR-34a was negatively significantly associated with PLCG1, miR-30a-5p was negatively significantly associated with PIK3R2, miR-27a was negatively significantly associated with GRB2, miR-302b was positively significantly associated with JAK1 and miR-520e was negatively significantly associated with JAK1, as well as miR-155 was positively significantly associated with JAK2 (Table 2). Two directions were found for the mRNA:miRNA relationship: negative and positive. The results revealed that some mRNAs had only one significant miRNA, while, some mRNAs had more than one significant miRNAs.

**Table 2:** **Significant miRNAs targeting the shared downstream molecules (***p*** < 0.05)**.

**mRNA**	**Significant miRNAs**	
	**Positive (+)**	**Negative (−)**
PLCG1		miR-34a
PIK3R2		miR-30a-5p
GRB2		miR-27a
JAK1	miR-302b	miR-520e
JAK2	miR-155	

### SVR model to determine the best weighted miRNA(s)

**Figure 2:**
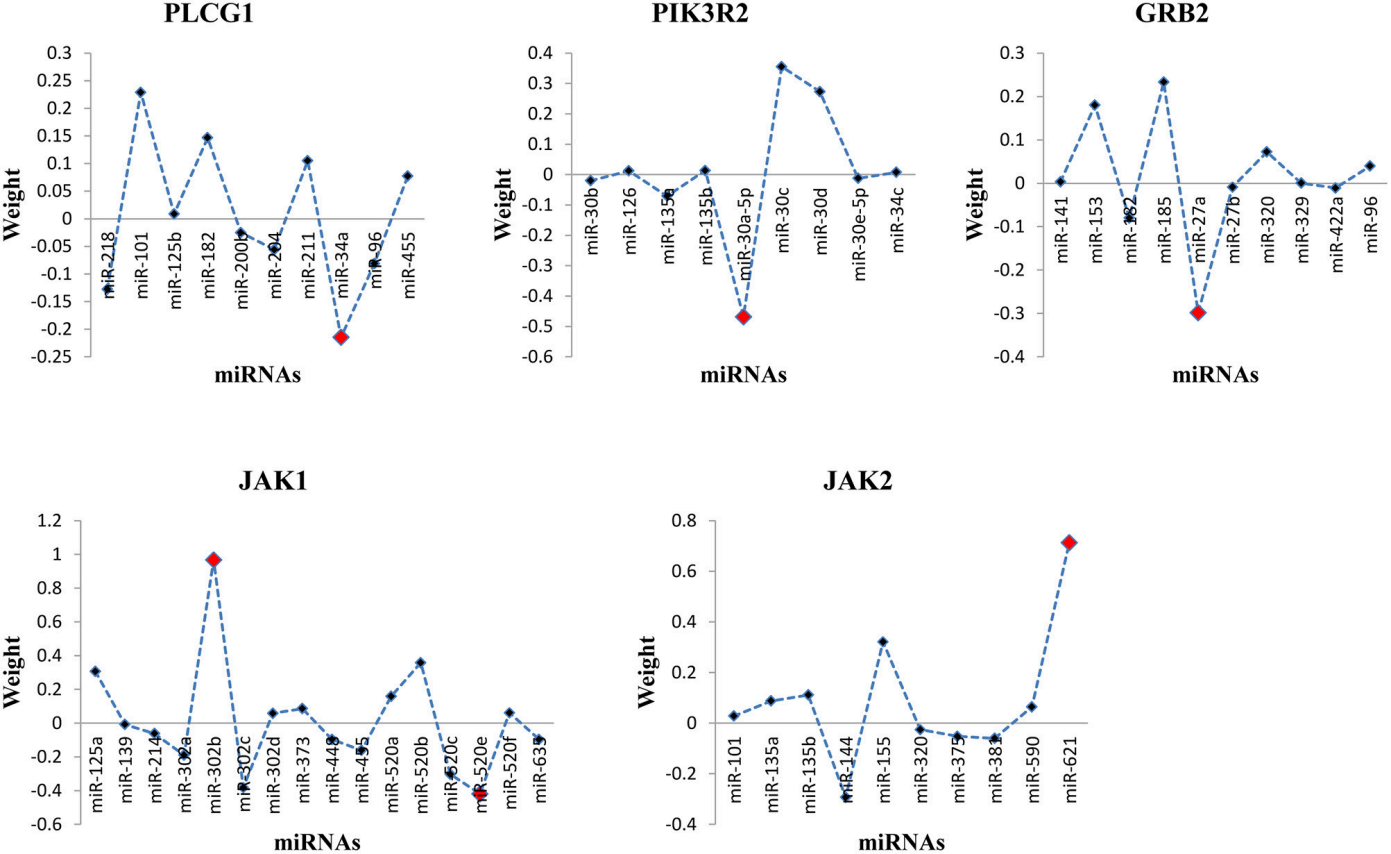
**Distribution of miRNA weights for the target mRNAs in the SVR model**. The red dot indicated the best weighted miRNA(s).

**Figure 3:**
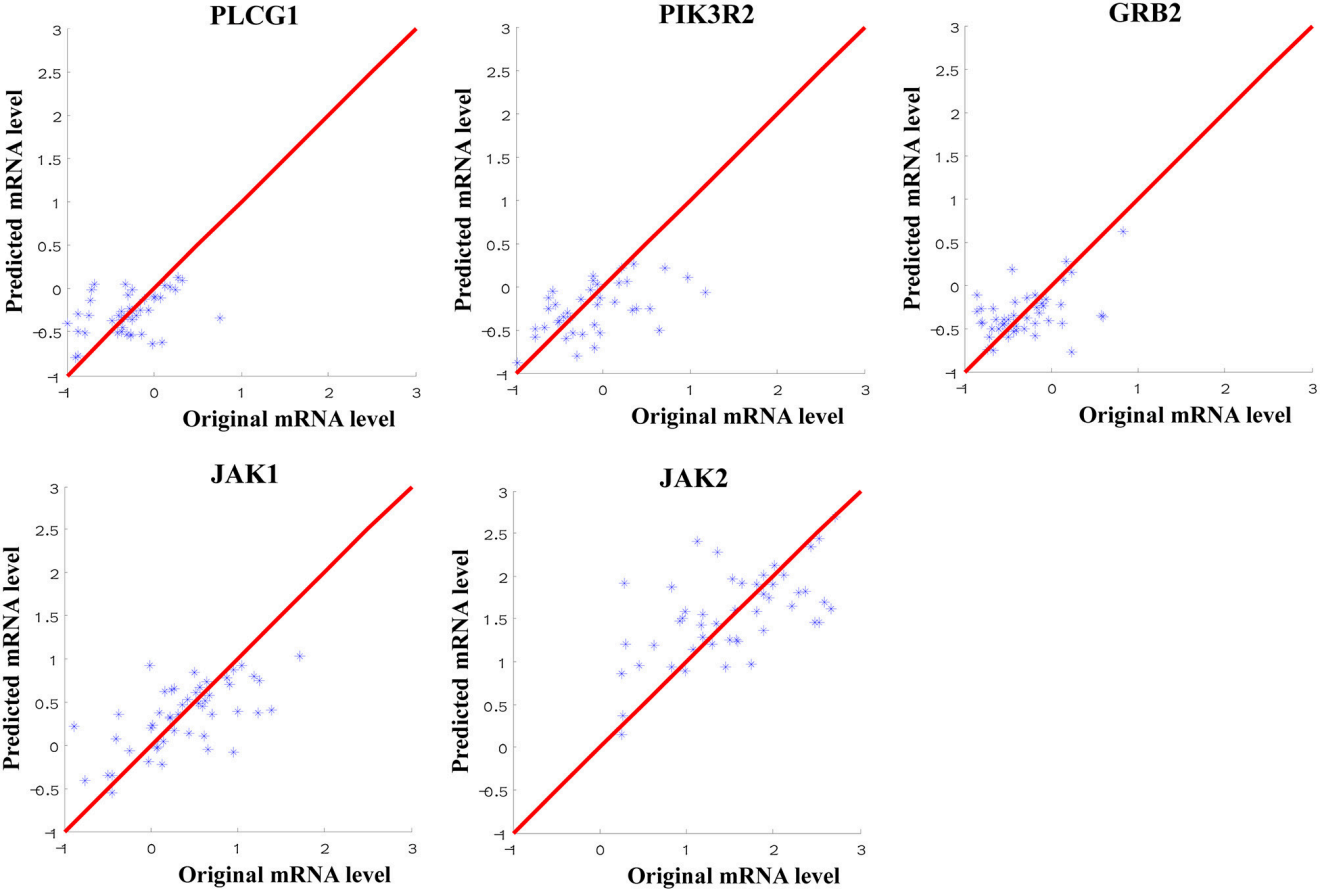
**Scatter diagram for the expression levels of the original mRNAs from microarray data and the predicted mRNAs from SVR models**. The red line referred to Y = X to indicate the location of the data points.

Through the SVR model manipulation, the linear regression coefficient of each miRNA was obtained, which represented the weight of each miRNA making the contribution to the expression levels of the target mRNAs. The results depicted (Figure 2): miR-34a had the largest negative weight in the PLCG1 model, miR-30a-5p had the largest negative weight in the PIK3R2 model, miR-27a had the largest negative weight in the GRB2 model, as well as miR-302b had the largest positive weight and miR-520e had the largest negative weight in the JAK1 model. The results were consistent with the findings of the multiple linear regression model that these best weighted miRNAs had the significant *p*-values, and the signs of the weights were also the same, indicating the same direction. A little bit difference was found in JAK2 model that miR-155 had the second largest positive weight. The weight of miR-621 was the largest. In the multiple linear regression model, the *p*-value for miR-621 was 0.057, nearly significant. Possibly, enlarging the sample size could polish the results. In order to prove the prediction efficiency of the SVR model, we plotted the scatter diagrams for the expression levels of the original mRNAs from the microarray data and the predicted mRNAs based on the constructed SVR model (Figure 3). From the results, we could demonstrate that the data points were concentrated around the red line Y = X, which means that the SVR method was capable of well modeling the relationship between mRNAs and the multiple miRNAs.

## Discussion

In this study, we investigated the direct EGFR downstream molecules in the EGFR signaling pathway shared with the other TKRs that may affect the drug resistance (Table 1, Figure 1). The statistically significant and the best weighted miRNAs regulating the mRNAs of these shared downstream molecules were further explored using the multiple linear regression and support vector regression models (Table 2, Figure 2). Multiple linear regression can obtain the linear regression equation to represent the relationship between the mRNA and the multiple miRNAs by minimizing the mean least square error. It can also indicate the significance of each miRNA coefficient from the point of statistical view. By contrast, SVR can model the associations between mRNAs and miRNAs through maximizing regression margin and minimizing error. The two introduced methods found the similar mRNA:miRNA associations. The potential miRNAs significantly affecting the expression of mRNAs from multiple regression analysis were further identified through the weights in the SVR model. Since one mRNA could be regulated by more than one miRNAs, multiple regression analysis is more suitable to simulate the *in vivo* environment and identity the significant miRNAs. Furthermore, SVR method could well model the associations between the mRNAs and the multiple miRNAs from the results (Figure 3). Our approaches using *in silico* analysis could demonstrate the important findings and infer the biological meaning before performing the experimental validation.

Our method could enable the identification of mRNAs that were co-regulated by multiple significant miRNAs and those that were targeted by only one significant miRNA, as well as the direction of the relationship. Both positive and negative relationships were found in our results (Table 2, Figure 2), such as the positive association between JAK1 and miR-302b, and the negative association between PLCG1 and miR-34a. Some molecules had more than one significant miRNAs, e.g., JAK1. Different interaction types may exist to affect the associations between mRNAs and miRNAs. The direct binding of miRNAs to the seed regions of mRNAs can influence the stability of mRNAs and the translation process from mRNAs to proteins (Androsavich et al., 2012). Since miRNA binding could degrade the target mRNAs, negative associations will be possibly found. Researchers have reported that some mRNAs may not be degraded when miRNAs bind to the seed regions. The bindings could lead to the accumulations of the inhibited mRNAs (Franco-Zorrilla et al., 2007; Ambs et al., 2008). This mechanism may result in the positive relationship between mRNAs and miRNAs. Moreover, the indirect interaction through the intermediators between them could also generate the positive associations.

The presence of other activated TKRs can mimic the function of EGFR by activating the shared signal transduction pathways to promote cell survival (Camp et al., 2005). Downstream molecule, phosphoinositide-3-kinase (PI3K), shared by both EGFR and one of these TKRs IGF-1R has been reported to affect the resistance to EGFR-TKIs (Camp et al., 2005). MiRNAs play important roles in the EGFR signaling pathway, therefore, they are remarkable for drug discovery in cancer therapy. To investigate the relationship between miRNAs and drug resistance makes the valuable contribution to improve the therapeutic efficacy of cancer. Since not every protein is suitable for drug target, specific manipulation of the undruggable proteins can be realized through the corresponding miRNAs targeting the mRNAs: miRNA inhibitors can upregulate one protein and miRNA mimics can silence one gene (Schmidt, 2014). People have tested a liposomal nanoparticle loaded with synthetic miRNA-34a mimics (MRX34) that against cancer cells when combined with the EGFR-TKI Erlotinib treatment (Bader, 2012; Zhao et al., 2014). The additive or synergistic effects of miRNAs together with another treatment provide a novel platform for treating resistant cancer cells (Wu, 2010). MiR-15b and miR-16 in combination with chemotherapy can induce the sensitivity of gastric cancer cells by inhibition of cyclin D1 (BCL1) (Zhao et al., 2008). Consequently, inducing the cancer cell sensitivity by miRNAs provides a novel approach for cancer treatment, at least *in vitro* (Wu, 2010).

The candidate miRNAs discovered in our study have been investigated in cancer research. MiR-34a has been identified as a novel prognostic marker in NSCLCs, making great contributions for evaluating the recurrence risk of patients and providing useful information for cancer treatment (Gallardo et al., 2009). Other researchers found that miR-34a can significantly reduce the tumor growth in an *in vivo* murine model and activate some major proteins involved in the apoptosis process, which may act as a suppressor of tumorigenesis (Welch et al., 2007; Tivnan et al., 2011). It has been reported that miR-30a might function as a metastasis suppressor by downregulating phosphatidylinositol-4,5-bisphosphate 3-kinase catalytic subunit delta (PIK3CD) in colorectal carcinoma to suppresses cell migration and invasion (Zhong et al., 2013). MiR-27a plays an important role in the regulation of drug resistance, indicating its potential property for therapeutic strategy in cancer cell lines (Zhu et al., 2008). MiR-155 has been explored as the well characterized signatures for breast, colon and lung cancers (Volinia et al., 2006). To our best knowledge, some of the identified miRNAs in our study have limited information for NSCLC research in the literatures, which may be the novel and important findings needed to be further exploration.

In summary, our study introduced the mRNA:miRNA multiple linear regression and SVR models to identify the significant and the best weighted miRNAs regulating the shared downstream molecules of EGFR and other TKRs that may affect the drug resistance. These candidate miRNAs can be regarded as the potential drug targets for NSCLC treatment, or combined with Gefitinib and Erlotinib treatment. Several methods have been established to identify the associations between miRNAs and mRNAs. One of the widely used methods is the application of gene set test (GST). In this method, the first step is to select the putative miRNA-mRNA pairs using the prediction databases. And then, the GST is performed to get the significant mRNAs for a given miRNA based on the microarray data (Subramanian et al., 2005; Efron and Tibshirani, 2007). Another method is the application of odds-ratio (OR) statistic (Jayaswal et al., 2009). If the significant associations are found between miRNA and its computationally predicted target mRNAs according to microarray expression levels, the miRNA is considered to have the regulatory function. These two mentioned methods can only identify the individual regulatory miRNAs, and ignore the behind biological meaning that multiple miRNAs may co-regulate a target mRNA simultaneously. Jayaswal et al. proposed a method to identify the mRNA:miRNA modules (Jayaswal et al., 2011). Firstly, to identify the miRNA and mRNA clusters using computationally predicted approach and expression data. Secondly, to obtain the statistically significant associations between miRNA and mRNA clusters based on the expression levels. This method only considers the significant cluster pairs, and do not take into account the weight of the multiple miRNAs on the same mRNAs. Our developed method incorporated these factors, and could identify the statistically significant and the best weighted miRNA(s) for a particular mRNA.

In the future, we will collect more public datasets to perform the similar analysis to polish our findings. Laboratory experimental validation will be done to explore the true effects of the identified miRNAs on drug resistance in NSCLCs, including Polymerase chain reaction (PCR) and wester-blot. Furthermore, knock-down of the other TKRs and the shared downstream molecules, as well as miRNA mimics/inhibitors experiments will be performed to support the hypothesis and the current findings.

## Author contributions

FW and LC initiated the project and participated in its design. FW, FM, and LC performed the multiple linear regression analyses and participated in the interpretation of data. FW and LW constructed the support vector regression (SVR) model. FM, LW, SW, and WC participated in the design and coordination of the study. FW was responsible for writing the manuscript. All the authors participated in the discussion and editing of the manuscript.

### Conflict of interest statement

The authors declare that the research was conducted in the absence of any commercial or financial relationships that could be construed as a potential conflict of interest.

## References

[B1] Agarwal V. Bell G. W. Nam J.-W. Bartel D. P. (2015). Predicting effective microRNA target sites in mammalian mRNAs. eLife 4:e05005. 10.7554/eLife.05005 26267216 PMC4532895

[B2] Al-Saleh K. Quinton C. Ellis P. M. (2012). Role of pemetrexed in advanced non-small-cell lung cancer: meta-analysis of randomized controlled trials, with histology subgroup analysis. Curr. Oncol. 19, e9–e15. 10.3747/co.19.891 22328848 PMC3267597

[B3] Ambs S. Prueitt R. L. Yi M. Hudson R. S. Howe T. M. Petrocca F.. (2008). Genomic profiling of microRNA and messenger RNA reveals deregulated microRNA expression in prostate cancer. Cancer Res. 68, 6162–6170. 10.1158/0008-5472.CAN-08-0144 18676839 PMC2597340

[B4] Androsavich J. R. Chau B. N. Bhat B. Linsley P. S. Walter N. G. (2012). Disease-linked microRNA-21 exhibits drastically reduced mRNA binding and silencing activity in healthy mouse liver. RNA 18, 1510–1526. 10.1261/rna.033308.112 22740638 PMC3404372

[B5] Arima C. Kajino T. Tamada Y. Imoto S. Shimada Y. Nakatochi M.. (2014). Lung adenocarcinoma subtypes definable by lung development-related miRNA expression profiles in association with clinicopathologic features. Carcinogenesis 35, 2224–2231. 10.1093/carcin/bgu127 24903339

[B6] Bader A. G. (2012). miR-34 - a microRNA replacement therapy is headed to the clinic. Front. Genet. 3:120. 10.3389/fgene.2012.00120 22783274 PMC3387671

[B7] Betel D. Koppal A. Agius P. Sander C. Leslie C. (2010). Comprehensive modeling of microRNA targets predicts functional non-conserved and non-canonical sites. Genome Biol. 11:R90. 10.1186/gb-2010-11-8-r90 20799968 PMC2945792

[B8] Breslin T. Krogh M. Peterson C. Troein C. (2005). Signal transduction pathway profiling of individual tumor samples. BMC Bioinformatics 6:163. 10.1186/1471-2105-6-163 15987529 PMC1184060

[B9] Camp E. R. Summy J. Bauer T. W. Liu W. Gallick G. E. Ellis L. M. (2005). Molecular mechanisms of resistance to therapies targeting the epidermal growth factor receptor. Clin. Cancer Res. 11, 397–405. 15671571

[B10] Efron B. Tibshirani R. (2007). On testing the significance of sets of genes. Ann. Appl. Statist. 1, 107–129. 10.1214/07-AOAS10115189895

[B11] Franco-Zorrilla J. M. Valli A. Todesco M. Mateos I. Puga M. I. Rubio-Somoza I.. (2007). Target mimicry provides a new mechanism for regulation of microRNA activity. Nat. Genet. 39, 1033–1037. 10.1038/ng2079 17643101

[B12] Gallardo E. Navarro A. Viñolas N. Marrades R. M. Diaz T. Gel B.. (2009). miR-34a as a prognostic marker of relapse in surgically resected non-small-cell lung cancer. Carcinogenesis 30, 1903–1909. 10.1093/carcin/bgp219 19736307

[B13] Griffiths-Jones S. Saini H. K. van Dongen S. Enright A. J. (2008). miRBase: tools for microRNA genomics. Nucleic Acids Res. 36, D154–D158. 10.1093/nar/gkm952 17991681 PMC2238936

[B14] Gunn S. R. (1998). Support Vector Machines for Classification and Regression. ISIS Technical Report 14, University of Southampton.

[B15] Indovina P. Marcelli E. Maranta P. Tarro G. (2011). Lung cancer proteomics: recent advances in biomarker discovery. Int. J. Proteomics 2011:726869. 10.1155/2011/726869 22229091 PMC3196861

[B16] Jayaswal V. Lutherborrow M. Ma D. D. Hwa Yang Y. (2009). Identification of microRNAs with regulatory potential using a matched microRNA-mRNA time-course data. Nucleic Acids Res. 37, e60. 10.1093/nar/gkp153 19295134 PMC2677888

[B17] Jayaswal V. Lutherborrow M. Ma D. D. Yang Y. H. (2011). Identification of microRNA-mRNA modules using microarray data. BMC Genomics 12:138. 10.1186/1471-2164-12-138 21375780 PMC3065435

[B18] Kapp A. V. Jeffrey S. S. Langerød A. Børresen-Dale A. L. Han W. Noh D. Y.. (2006). Discovery and validation of breast cancer subtypes. BMC Genomics 7:231. 10.1186/1471-2164-7-231 16965636 PMC1574316

[B19] Kim E. S. Khuri F. R. Herbst R. S. (2001). Epidermal growth factor receptor biology (IMC-C225). Curr. Opin. Oncol. 13, 506–513. 10.1097/00001622-200111000-00014 11673692

[B20] Krek A. Grün D. Poy M. N. Wolf R. Rosenberg L. Epstein E. J.. (2005). Combinatorial microRNA target predictions. Nat. Genet. 37, 495–500. 10.1038/ng1536 15806104

[B21] Kumarakulasinghe N. B. van Zanwijk N. Soo R. A. (2015). Molecular targeted therapy in the treatment of advanced stage non-small cell lung cancer (NSCLC). Respirology 20, 370–378. 10.1111/resp.12490 25689095

[B22] Lin P. Y. Yu S. L. Yang P. C. (2010). MicroRNA in lung cancer. Br. J. Cancer 103, 1144–1148. 10.1038/sj.bjc.6605901 20859290 PMC2967070

[B23] Lu J. Getz G. Miska E. A. Alvarez-Saavedra E. Lamb J. Peck D.. (2005). MicroRNA expression profiles classify human cancers. Nature 435, 834–838. 10.1038/nature03702 15944708

[B24] Ma L. Teruya-Feldstein J. Weinberg R. A. (2007). Tumour invasion and metastasis initiated by microRNA-10b in breast cancer. Nature 449, 682–688. 10.1038/nature06174 17898713

[B25] Schmidt M. F. (2014). Drug target miRNAs: chances and challenges. Trends Biotechnol. 32, 578–585. 10.1016/j.tibtech.2014.09.002 25304465

[B26] Smola A. Vapnik V. (1997). Support vector regression machines. Adv. Neural Inf. Process. Syst. 9, 155–161. 19325729

[B27] Subramanian A. Tamayo P. Mootha V. K. Mukherjee S. Ebert B. L. Gillette M. A.. (2005). Gene set enrichment analysis: a knowledge-based approach for interpreting genome-wide expression profiles. Proc. Natl. Acad. Sci. U.S.A. 102, 15545–15550. 10.1073/pnas.0506580102 16199517 PMC1239896

[B28] Tivnan A. Tracey L. Buckley P. G. Alcock L. C. Davidoff A. M. Stallings R. L. (2011). MicroRNA-34a is a potent tumor suppressor molecule *in vivo* in neuroblastoma. BMC Cancer 11:33. 10.1186/1471-2407-11-33 21266077 PMC3038978

[B29] Volinia S. Calin G. A. Liu C. G. Ambs S. Cimmino A. Petrocca F.. (2006). A microRNA expression signature of human solid tumors defines cancer gene targets. Proc. Natl. Acad. Sci. U.S.A. 103, 2257–2261. 10.1073/pnas.0510565103 16461460 PMC1413718

[B30] Wang F. Chan L. W. Law H. K. Cho W. C. Tang P. Yu J.. (2014a). Exploring microRNA-mediated alteration of EGFR signaling pathway in non-small cell lung cancer using an mRNA:miRNA regression model supported by target prediction databases. Genomics 104, 504–511. 10.1016/j.ygeno.2014.09.004 25257143

[B31] Wang F. Wong S. C. Chan L. W. Cho W. C. Yip S. P. Yung B. Y. (2014b). Multiple regression analysis of mRNA-miRNA associations in colorectal cancer pathway. Biomed Res. Int. 2014:676724. 10.1155/2014/676724 24895601 PMC4033420

[B32] Wang H. Wu S. Zhao L. Zhao J. Liu J. Wang Z. (2015). Clinical use of microRNAs as potential non-invasive biomarkers for detecting non-small cell lung cancer: a meta-analysis. Respirology 20, 56–65. 10.1111/resp.12444 25440223

[B33] Webster R. J. Giles K. M. Price K. J. Zhang P. M. Mattick J. S. Leedman P. J. (2009). Regulation of epidermal growth factor receptor signaling in human cancer cells by microRNA-7. J. Biol. Chem. 284, 5731–5741. 10.1074/jbc.M804280200 19073608

[B34] Weiss G. J. Bemis L. T. Nakajima E. Sugita M. Birks D. K. Robinson W. A.. (2008). EGFR regulation by microRNA in lung cancer: correlation with clinical response and survival to gefitinib and EGFR expression in cell lines. Ann. Oncol. 19, 1053–1059. 10.1093/annonc/mdn006 18304967

[B35] Welch C. Chen Y. Stallings R. L. (2007). MicroRNA-34a functions as a potential tumor suppressor by inducing apoptosis in neuroblastoma cells. Oncogene 26, 5017–5022. 10.1038/sj.onc.1210293 17297439

[B36] Wong N. Wang X. (2015). miRDB: an online resource for microRNA target prediction and functional annotations. Nucleic Acids Res. 43, D146–D152. 10.1093/nar/gku1104 25378301 PMC4383922

[B37] Wu W. (2010). MicroRNA: potential targets for the development of novel drugs? Drugs R D 10, 1–8. 10.2165/11537800-000000000-00000 20509710 PMC3585691

[B38] Zandberga E. Kozirovskis V. Abols A. Andrejeva D. Purkalne G. Line A. (2013). Cell-free microRNAs as diagnostic, prognostic, and predictive biomarkers for lung cancer. Genes Chromosomes Cancer 52, 356–369. 10.1002/gcc.22032 23404859

[B39] Zhang B. Farwell M. A. (2008). microRNAs: a new emerging class of players for disease diagnostics and gene therapy. J. Cell. Mol. Med. 12, 3–21. 10.1111/j.1582-4934.2007.00196.x 18088390 PMC3823469

[B40] Zhang Z. Lee J. C. Lin L. Olivas V. Au V. LaFramboise T.. (2012). Activation of the AXL kinase causes resistance to EGFR-targeted therapy in lung cancer. Nat. Genet. 44, 852–860. 10.1038/ng.2330 22751098 PMC3408577

[B41] Zhao J. Kelnar K. Bader A. G. (2014). In-depth analysis shows synergy between erlotinib and miR-34a. PLoS ONE 9:e89105. 10.1371/journal.pone.0089105 24551227 PMC3925231

[B42] Zhao J. J. Lin J. Yang H. Kong W. He L. Ma X.. (2008). MicroRNA-221/222 negatively regulates estrogen receptor alpha and is associated with tamoxifen resistance in breast cancer. J. Biol. Chem. 283, 31079–31086. 10.1074/jbc.M806041200 18790736 PMC2576549

[B43] Zhong M. Bian Z. Wu Z. (2013). miR-30a suppresses cell migration and invasion through downregulation of PIK3CD in colorectal carcinoma. Cell. Physiol. Biochem. 31, 209–218. 10.1159/000343362 23486085

[B44] Zhu H. Wu H. Liu X. Evans B. R. Medina D. J. Liu C. G.. (2008). Role of MicroRNA miR-27a and miR-451 in the regulation of MDR1/P-glycoprotein expression in human cancer cells. Biochem. Pharmacol. 76, 582–588. 10.1016/j.bcp.2008.06.007 18619946 PMC2628586

